# (2,2′-Bi­pyridine-κ^2^ *N*,*N*′)tetra­kis­(dimethyl sulfoxide-κ*O*)copper(II) bis­(perchlorate)

**DOI:** 10.1107/S1600536813018485

**Published:** 2013-07-10

**Authors:** Turganbay Iskenderov

**Affiliations:** a Department of Chemistry, National Taras, Shevchenko University, Volodymyrska, Str. 64, 01601 Kyiv, Ukraine

## Abstract

The title compound, [Cu(C_2_H_6_OS)_4_(C_10_H_8_N_2_)](ClO_4_)_2_, contains a Cu^II^ ion with a distorted octa­hedral coordination environment, bonded by four O atoms of the monodentate dimethyl sulfoxide ligands and two N atoms of the bidentate chelating 2,2′-bi­pyridine ligand. The equatorial Cu—N and Cu—O bond lengths are in the range 1.979 (2)-1.998 (3) Å. The axial Cu—O bond distances are 2.365 (2) and 2.394 (2) Å. In the crystal, the complex cations and perchlorate anions are connected by numerous C—H⋯O hydrogen bonds, which leads to additional stabilization of the structure. The perchlorate anion is disordered over two sets of sites with a 0.716 (3):0.284 (3) occupancy ratio.

## Related literature

For applications of the 2,2′-bipyridyl ligand, see: Fritsky *et al.* (2004 ▶, 2006 ▶); Kanderal *et al.* (2005 ▶). For related structures, see: Fritsky *et al.* (1998 ▶, 2000 ▶); Moroz *et al.* (2010 ▶, 2012 ▶); Sliva *et al.* (1997 ▶); Świątek-Kozłowska *et al.* (2000 ▶, 2002 ▶); Iskenderov *et al.* (2009 ▶); Golenya *et al.* (2012*a* ▶). For the synthesis, see: Golenya *et al.* (2012*b* ▶).
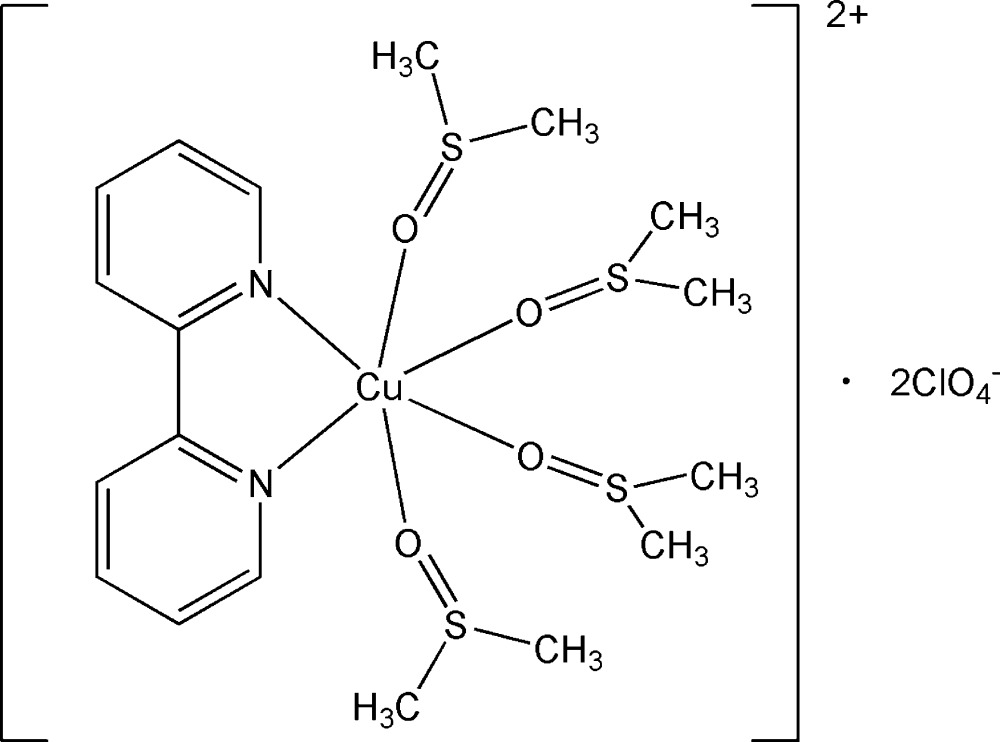


## Experimental

### 

#### Crystal data

[Cu(C_2_H_6_OS)_4_(C_10_H_8_N_2_)](ClO_4_)_2_
*M* *_r_* = 731.14
Monoclinic, 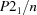

*a* = 10.8050 (5) Å
*b* = 11.6470 (5) Å
*c* = 24.5210 (8) Å
β = 94.984 (5)°
*V* = 3074.2 (2) Å^3^
*Z* = 4
Mo *K*α radiation
μ = 1.21 mm^−1^
*T* = 120 K
0.33 × 0.23 × 0.12 mm

#### Data collection

Nonius KappaCCD diffractometer
Absorption correction: multi-scan (*MULABS*; Blessing, 1995 ▶) *T*_min_ = 0.747, *T*_max_ = 0.862
40246 measured reflections
13140 independent reflections
6565 reflections with *I* > 2σ(*I*)
*R*_int_ = 0.074

#### Refinement

*R*[*F* ^2^ > 2σ(*F* ^2^)] = 0.075
*wR*(*F* ^2^) = 0.160
*S* = 1.07
13140 reflections
362 parameters
10 restraints
H-atom parameters constrained
Δρ_max_ = 1.21 e Å^−3^
Δρ_min_ = −0.84 e Å^−3^

### 

Data collection: *COLLECT* (Nonius, 2000 ▶); cell refinement: *DENZO*/*SCALEPACK* (Otwinowski & Minor, 1997 ▶); data reduction: *DENZO*/*SCALEPACK*; program(s) used to solve structure: *SIR2004* (Burla *et al.*, 2005 ▶); program(s) used to refine structure: *SHELXL97* (Sheldrick, 2008 ▶); molecular graphics: *DIAMOND* (Brandenburg, 2009 ▶); software used to prepare material for publication: *SHELXL97*.

## Supplementary Material

Crystal structure: contains datablock(s) I, New_Global_Publ_Block. DOI: 10.1107/S1600536813018485/nr2046sup1.cif

Structure factors: contains datablock(s) I. DOI: 10.1107/S1600536813018485/nr2046Isup2.hkl

Additional supplementary materials:  crystallographic information; 3D view; checkCIF report

## Figures and Tables

**Table 1:** Hydrogen-bond geometry (Å, °)

*D*—H⋯*A*	*D*—H	H⋯*A*	*D*⋯*A*	*D*—H⋯*A*
C1—H1⋯O2	0.95	2.52	3.046 (4)	115
C6—H6⋯O8^i^	0.95	2.47	3.255 (5)	140
C8—H8⋯O3	0.95	2.42	2.946 (4)	115
C4—H4⋯O1^ii^	0.95	2.35	3.219 (4)	151
C16—H16*B*⋯O5^iii^	0.98	2.56	3.411 (5)	145
C17—H17*B*⋯O5^iii^	0.98	2.40	3.306 (5)	153
C15—H15*C*⋯O7^iv^	0.98	2.51	3.320 (5)	140

Symmetry codes: (i) 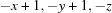; (ii) 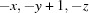; (iii) 
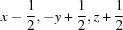
; (iv) 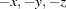.

**Fig. 1.**
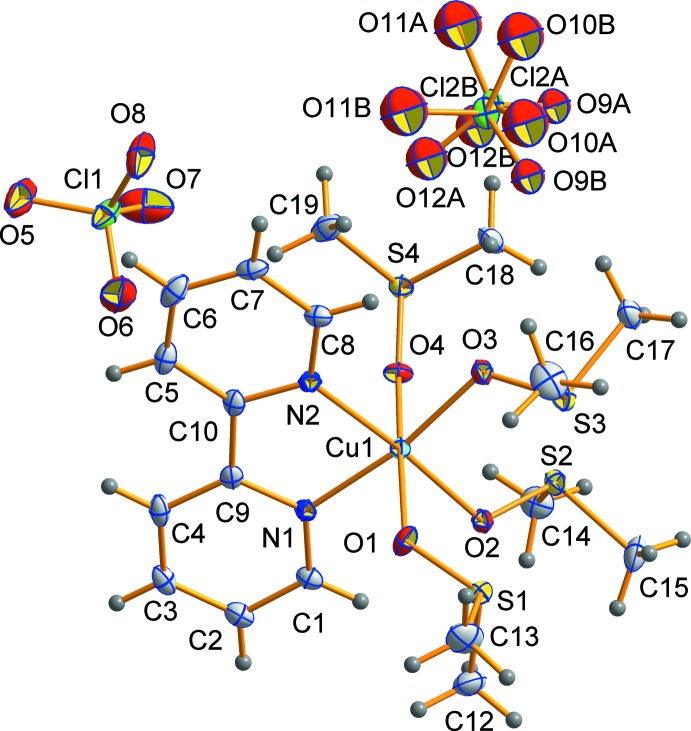
A view of the title compound, with displacement ellipsoids shown at the 40% probability level. Both orientations for the disordered perchlorate anion are shown. H atoms are drawn as spheres of arbitrary radii.

**Fig. 2.**
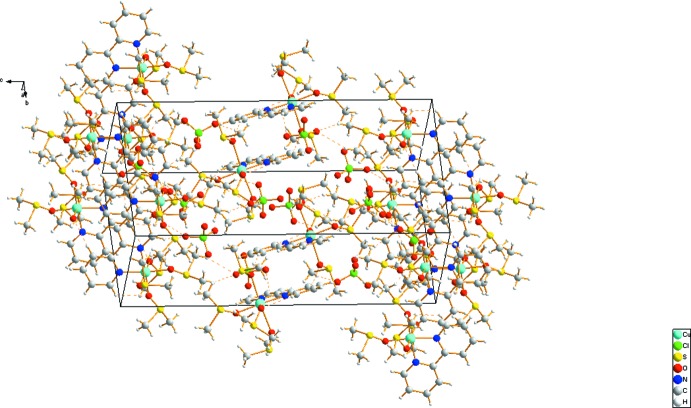
Crystal packing of the title compound. Hydrogen bonds are indicated by dashed lines. Only major orientation is shown for the disordered perchlorate anion.

## supplementary crystallographic information

### Comment

2,2'-Bipyridyl (2,2'-bipy) is a well known neutral bidentate ligand which is widely used in coordination chemistry, in particular, for the preparation of mixed ligand complexes (Fritsky *et al.*, 2004; Kanderal *et al.*, 2005). It is also often used in the synthesis of discrete polynuclear complexes in order to prevent formation of coordination polymers by blocking a certain number of vacant sites in the coordination sphere of a metal ion (Fritsky *et al.*, 2006). The title compound was obtained unintentionally during an attempt to synthesize a mixed-ligand complex of Cu^II^ with pyridine-2-hydroxamic acid in the framework of our project on study of metal complexes of heterocyclic hydroxamic acids (Świątek-Kozłowska *et al.*, 2002; Golenya *et al.*, 2012*a*). The crystal structure of the title complex has not hitherto been reported.

The title compound, [Cu(C_10_H_8_N_2_)(C_2_H_6_OS)_4_](ClO_4_)_2_, consists of a cationic Cu^2+^ complex and two perchlorate anions (Fig. 1). The central atom is in distorted octahedral environment formed by two nitrogen donor atoms of bidentately coordinated molecule of 2,2'-bipyridine and by four oxygen atoms of dimethyl sulfoxide molecules. The equatorial plane of the coordination polyhedron is consisting of two nitrogen atoms of 2,2'-bipyridyl and two oxygen atoms of two dimethyl sulfoxide ligands. The axial positions are occupied by oxygen atoms of other two dimethyl sulfoxide ligands. The equatorial Cu—N and Cu—O bond lengths are in the range 1.979 (2) – 1.998 (3) Å. The axial Cu—O bond distances are 2.365 (2) Å and 2.394 (2) Å. The coordination bond lengths Cu—N and Cu—O are typical for distorted octahedral Cu^II^ complexes with the nitrogen and oxygen donors (Fritsky *et al.*, 1998; Świątek-Kozłowska *et al.*, 2000; Sliva *et al.*, 1997). The N2—Cu1—N1 bite angle is decreased to 81.45 (11)°, which is a consequence of the formation of five-membered chelate ring. The C—N and C—C bond lengths in the pyridine rings are normal for 2-substituted pyridine derivatives (Fritsky *et al.*, 2000; Iskenderov *et al.*, 2009; Moroz *et al.*, 2010; Moroz *et al.*, 2012).

In the crystal structure, the complex cations and perchlorate anions are connected by numerous intermolecular C—H···O hydrogen bonds, in which the aromatic and methyl carbon atoms act as donors while the perchlorate oxygen atoms act as acceptors, which contribute to the stabilization of the structure (Fig. 2). There are also stacking interactions between the aromatic rings of the 2,2'-bipyridine molecules belonging to the neighboring complex molecules with shortest non-covalent contacts C(2)···C(6) (-*x*, 1 - *y*, -*z*) = 3.369 (5) and C(4)···C(10) (-*x*, 1 - *y*, -*z*) = 3.390 (5) Å (Fig. 2).

### Experimental

The title compound was obtained unexpectedly during an attempt to synthesize a mixed-ligand complex of Cu^II^ with pyridine-2-hydroxamic acid (Golenya *et al.*, 2012*b*) in aqueous methanol solution. Cu(ClO_4_)_2_.6H_2_O in methanol (0.0370 g, 0.1 mmol) was added to solution of 2,2'-bipyridine (0.156 g, 1 mmol) and pyridine-2-hydroxamic acid (0.069 g, 0.5 mmol) in methanol (7 ml), afterwards the obtained transparent blue solution left for evaporation in the air at ambient temperature. The obtained dry solid residue was dissolved in 5 ml DMSO and set for crystallization by slow diffusion of 2-propanol vapors at room temperature. During 12 h the blue crystals suitable for X-ray analysis were obtained. They were filtered off, washed with 2-propanol and dried in the air. Yield: 73%. Elemental analysis calc. (%) for C_18_H_32_Cl_2_CuN_2_O_12_S_4_: C 29.63; H 4.42; N 3.84; found: C 29.78; H 4.60; N 3.63.

### Refinement

The C—H atoms were positioned geometrically and refined as riding atoms, with C—H = 0.95(CH), 0.98(CH_3_) and U_iso_ = 1.2 or 1.5 U_eq_(parent atom) for CH and CH_3_, respectively. One of the perchlorate anions were found to be disordered over two positions with occupancy factors 0.716/0.284. All atoms were refined with anisotropic displacement parameters except the oxygen atoms of the disordered perchlorate which were refined isotropically. Cl—O bond distances and O···O separations of both (minor and major) fractions of the disordered perchlorate anion as well as the ordered perchlorate (as a model) were restrained to have identical values. For each corresponding pair of the oxygen atoms of the disordered perchlorate anion, the isotropic displacement parameters were restrained to have identical values.

### Crystal data

**Table:**

[Cu(C_2_H_6_OS)_4_(C_10_H_8_N_2_)](ClO_4_)_2_	*F*(000) = 1508
*M**_r_* = 731.14	*D*_x_ = 1.580 Mg m^−^^3^
Monoclinic, *P*2_1_/*n*	Mo *K*α radiation, λ = 0.71073 Å
Hall symbol: -P 2yn	Cell parameters from 2567 reflections
*a* = 10.8050 (5) Å	θ = 3.0–25.5°
*b* = 11.6470 (5) Å	µ = 1.21 mm^−^^1^
*c* = 24.5210 (8) Å	*T* = 120 K
β = 94.984 (5)°	Block, blue
*V* = 3074.2 (2) Å^3^	0.33 × 0.23 × 0.12 mm
*Z* = 4	

### Data collection

**Table:**

Nonius KappaCCD diffractometer	13140 independent reflections
Radiation source: fine-focus sealed tube	6565 reflections with *I* > 2σ(*I*)
Horizontally mounted graphite crystal monochromator	*R*_int_ = 0.074
Detector resolution: 9 pixels mm^-1^	θ_max_ = 36.7°, θ_min_ = 3.0°
φ scans and ω scans with κ offset	*h* = −13→17
Absorption correction: multi-scan (*MULABS*; Blessing, 1995)	*k* = −15→19
*T*_min_ = 0.747, *T*_max_ = 0.862	*l* = −40→31
40246 measured reflections	

### Refinement

**Table:**

Refinement on *F*^2^	Primary atom site location: structure-invariant direct methods
Least-squares matrix: full	Secondary atom site location: difference Fourier map
*R*[*F*^2^ > 2σ(*F*^2^)] = 0.075	Hydrogen site location: inferred from neighbouring sites
*wR*(*F*^2^) = 0.160	H-atom parameters constrained
*S* = 1.07	*w* = 1/[σ^2^(*F*_o_^2^) + (0.0589*P*)^2^] where *P* = (*F*_o_^2^ + 2*F*_c_^2^)/3
13140 reflections	(Δ/σ)_max_ < 0.001
362 parameters	Δρ_max_ = 1.21 e Å^−^^3^
10 restraints	Δρ_min_ = −0.84 e Å^−^^3^

### Special details

**Table:**

Experimental. The C—H atoms were positioned geometrically and refined as riding atoms, with C—H = 0.95(CH), 0.98(CH_3_) and U_iso_ = 1.2 or 1.5 U_eq_(parent atom) for CH and CH_3_, respectively. One of the perchlorate anions were found to be disordered over two positions with occupancy factors 0.716/0.284. All atoms were refined with anisotropic displacement parameters except the oxygen atoms of the disordered perchlorate which were refined isotropically. Cl—O bond distances and O···O separations of both (minor and major) fractions of the disordered perchlorate anion as well as the ordered perchlorate (as a model) were restrained to have identical values. For each corresponding pair of the oxygen atoms of the disordered perchlorate anion, the isotropic displacement parameters were restrained to have identical values.
Geometry. All esds (except the esd in the dihedral angle between two l.s. planes) are estimated using the full covariance matrix. The cell esds are taken into account individually in the estimation of esds in distances, angles and torsion angles; correlations between esds in cell parameters are only used when they are defined by crystal symmetry. An approximate (isotropic) treatment of cell esds is used for estimating esds involving l.s. planes.
Refinement. Refinement of F^2^ against ALL reflections. The weighted R-factor wR and goodness of fit S are based on F^2^, conventional R-factors R are based on F, with F set to zero for negative F^2^. The threshold expression of F^2^ > 2sigma(F^2^) is used only for calculating R-factors(gt) etc. and is not relevant to the choice of reflections for refinement. R-factors based on F^2^ are statistically about twice as large as those based on F, and R- factors based on ALL data will be even larger.

### Fractional atomic coordinates and isotropic or equivalent isotropic displacement parameters (Å^2^)

**Table:**

	*x*	*y*	*z*	*U*_iso_*/*U*_eq_	Occ. (<1)
Cu1	0.01095 (4)	0.25254 (3)	0.093944 (15)	0.01663 (10)	
S1	−0.22872 (8)	0.37353 (8)	0.15620 (4)	0.0260 (2)	
S2	−0.10117 (8)	0.03071 (7)	0.13267 (3)	0.02201 (19)	
S3	0.02546 (8)	0.21930 (7)	0.22086 (3)	0.02254 (19)	
S4	0.24417 (8)	0.03374 (7)	0.06730 (3)	0.02011 (18)	
O1	−0.1237 (2)	0.4013 (2)	0.12111 (10)	0.0256 (5)	
O2	−0.1282 (2)	0.14612 (18)	0.10375 (9)	0.0185 (5)	
O3	0.0926 (2)	0.22831 (19)	0.16856 (9)	0.0224 (5)	
O4	0.1196 (2)	0.0936 (2)	0.06162 (9)	0.0225 (5)	
Cl1	0.46540 (9)	0.22816 (8)	−0.07645 (4)	0.0300 (2)	
O5	0.5215 (3)	0.2784 (3)	−0.12148 (12)	0.0607 (10)	
O6	0.3432 (3)	0.2715 (2)	−0.07428 (13)	0.0490 (8)	
O7	0.4600 (3)	0.1059 (3)	−0.08316 (15)	0.0599 (10)	
O8	0.5387 (3)	0.2541 (3)	−0.02657 (11)	0.0535 (9)	
Cl2_1	0.4834 (4)	0.2572 (3)	0.25477 (14)	0.0236 (7)	0.716 (3)
O9_1	0.4557 (4)	0.1434 (3)	0.26534 (16)	0.0426 (10)*	0.716 (3)
O10_1	0.4453 (6)	0.3286 (5)	0.2980 (2)	0.0806 (16)*	0.716 (3)
O11_1	0.6132 (5)	0.2703 (5)	0.2537 (2)	0.0843 (17)*	0.716 (3)
O12_1	0.4289 (5)	0.2943 (4)	0.20296 (19)	0.0643 (13)*	0.716 (3)
Cl2_2	0.4738 (9)	0.2835 (7)	0.2563 (4)	0.0217 (17)	0.284 (3)
O9_2	0.3653 (8)	0.3114 (8)	0.2807 (4)	0.0426 (10)*	0.284 (3)
O10_2	0.5733 (10)	0.2666 (10)	0.2973 (5)	0.0806 (16)*	0.284 (3)
O11_2	0.5087 (11)	0.3809 (9)	0.2254 (5)	0.0843 (17)*	0.284 (3)
O12_2	0.4559 (10)	0.1942 (8)	0.2174 (4)	0.0643 (13)*	0.284 (3)
N1	−0.0439 (2)	0.2824 (2)	0.01530 (11)	0.0165 (6)	
N2	0.1453 (2)	0.3611 (2)	0.07722 (10)	0.0169 (6)	
C1	−0.1461 (3)	0.2403 (3)	−0.01254 (13)	0.0199 (7)	
H1	−0.2011	0.1935	0.0060	0.024*	
C2	−0.1736 (3)	0.2635 (3)	−0.06822 (14)	0.0235 (7)	
H2	−0.2471	0.2338	−0.0872	0.028*	
C3	−0.0930 (3)	0.3302 (3)	−0.09514 (13)	0.0229 (7)	
H3	−0.1093	0.3455	−0.1331	0.028*	
C4	0.0120 (3)	0.3746 (3)	−0.06631 (13)	0.0227 (8)	
H4	0.0682	0.4214	−0.0841	0.027*	
C5	0.2357 (3)	0.4606 (3)	0.00510 (14)	0.0251 (8)	
H5	0.2326	0.4815	−0.0324	0.030*	
C6	0.3321 (4)	0.4966 (3)	0.04112 (15)	0.0292 (9)	
H6	0.3967	0.5425	0.0287	0.035*	
C7	0.3345 (3)	0.4653 (3)	0.09585 (15)	0.0243 (8)	
H7	0.4001	0.4900	0.1215	0.029*	
C8	0.2394 (3)	0.3976 (3)	0.11236 (14)	0.0206 (7)	
H8	0.2406	0.3762	0.1498	0.025*	
C9	0.0340 (3)	0.3495 (3)	−0.01083 (13)	0.0182 (7)	
C10	0.1433 (3)	0.3933 (3)	0.02420 (13)	0.0183 (7)	
C12	−0.3584 (3)	0.3309 (4)	0.11044 (16)	0.0356 (9)	
H12A	−0.3773	0.3915	0.0832	0.053*	
H12B	−0.4307	0.3181	0.1312	0.053*	
H12C	−0.3382	0.2597	0.0918	0.053*	
C13	−0.2864 (4)	0.5103 (4)	0.17568 (17)	0.0423 (11)	
H13A	−0.2233	0.5490	0.2003	0.063*	
H13B	−0.3620	0.4994	0.1945	0.063*	
H13C	−0.3055	0.5575	0.1429	0.063*	
C14	−0.1260 (4)	−0.0737 (3)	0.08036 (15)	0.0320 (9)	
H14A	−0.2080	−0.0621	0.0608	0.048*	
H14B	−0.1217	−0.1506	0.0967	0.048*	
H14C	−0.0619	−0.0661	0.0547	0.048*	
C15	−0.2366 (4)	0.0083 (4)	0.16771 (16)	0.0364 (10)	
H15A	−0.2396	0.0655	0.1969	0.055*	
H15B	−0.2342	−0.0689	0.1837	0.055*	
H15C	−0.3106	0.0159	0.1419	0.055*	
C16	0.0575 (4)	0.3504 (3)	0.25593 (16)	0.0404 (10)	
H16A	0.1473	0.3645	0.2591	0.061*	
H16B	0.0282	0.3458	0.2926	0.061*	
H16C	0.0148	0.4133	0.2355	0.061*	
C17	0.1207 (4)	0.1252 (3)	0.26321 (15)	0.0332 (9)	
H17A	0.1133	0.0471	0.2485	0.050*	
H17B	0.0939	0.1263	0.3004	0.050*	
H17C	0.2074	0.1504	0.2642	0.050*	
C18	0.2790 (4)	0.0049 (4)	0.13850 (14)	0.0354 (10)	
H18A	0.2735	0.0762	0.1593	0.053*	
H18B	0.3632	−0.0265	0.1446	0.053*	
H18C	0.2192	−0.0509	0.1506	0.053*	
C19	0.3605 (3)	0.1404 (3)	0.06048 (17)	0.0324 (9)	
H19A	0.3422	0.1819	0.0260	0.049*	
H19B	0.4420	0.1034	0.0606	0.049*	
H19C	0.3612	0.1944	0.0911	0.049*	

### Atomic displacement parameters (Å^2^)

**Table:**

	*U*^11^	*U*^22^	*U*^33^	*U*^12^	*U*^13^	*U*^23^
Cu1	0.0172 (2)	0.01757 (19)	0.01494 (18)	−0.00310 (17)	0.00027 (14)	0.00218 (16)
S1	0.0200 (4)	0.0336 (5)	0.0251 (4)	0.0051 (4)	0.0070 (4)	0.0087 (4)
S2	0.0234 (5)	0.0188 (4)	0.0229 (4)	−0.0060 (3)	−0.0037 (3)	0.0044 (3)
S3	0.0262 (5)	0.0238 (4)	0.0173 (4)	−0.0015 (4)	0.0005 (3)	−0.0019 (3)
S4	0.0205 (4)	0.0190 (4)	0.0211 (4)	0.0024 (3)	0.0030 (3)	0.0007 (3)
O1	0.0245 (13)	0.0220 (13)	0.0321 (14)	−0.0021 (10)	0.0139 (11)	−0.0001 (10)
O2	0.0199 (12)	0.0161 (11)	0.0193 (11)	−0.0009 (9)	0.0007 (9)	0.0031 (9)
O3	0.0237 (13)	0.0249 (13)	0.0177 (11)	−0.0068 (10)	−0.0023 (10)	0.0041 (9)
O4	0.0174 (12)	0.0230 (12)	0.0267 (13)	0.0047 (10)	−0.0001 (10)	−0.0016 (10)
Cl1	0.0255 (5)	0.0340 (5)	0.0313 (5)	−0.0103 (4)	0.0071 (4)	−0.0072 (4)
O5	0.0444 (19)	0.113 (3)	0.0248 (15)	−0.0251 (19)	0.0071 (14)	0.0041 (17)
O6	0.0458 (19)	0.0425 (18)	0.061 (2)	0.0143 (15)	0.0204 (16)	0.0078 (15)
O7	0.048 (2)	0.0341 (17)	0.093 (3)	0.0076 (15)	−0.0144 (18)	−0.0270 (17)
O8	0.065 (2)	0.067 (2)	0.0276 (15)	−0.0458 (18)	0.0013 (14)	−0.0026 (15)
Cl2_1	0.0311 (11)	0.0185 (16)	0.0215 (9)	−0.0024 (11)	0.0034 (7)	0.0011 (9)
Cl2_2	0.034 (3)	0.012 (3)	0.020 (2)	0.001 (2)	0.0045 (18)	−0.0071 (19)
N1	0.0179 (14)	0.0133 (13)	0.0187 (13)	0.0033 (10)	0.0034 (11)	0.0018 (10)
N2	0.0151 (14)	0.0166 (13)	0.0190 (13)	0.0013 (11)	0.0016 (11)	−0.0001 (11)
C1	0.0211 (17)	0.0176 (16)	0.0206 (16)	0.0023 (14)	−0.0009 (13)	−0.0018 (13)
C2	0.0243 (18)	0.0236 (18)	0.0217 (16)	0.0047 (15)	−0.0028 (14)	−0.0014 (14)
C3	0.032 (2)	0.0213 (17)	0.0151 (16)	0.0091 (15)	0.0024 (14)	−0.0009 (13)
C4	0.034 (2)	0.0160 (16)	0.0196 (17)	0.0054 (14)	0.0092 (15)	0.0030 (13)
C5	0.031 (2)	0.0203 (18)	0.0255 (18)	−0.0012 (15)	0.0129 (16)	0.0001 (14)
C6	0.030 (2)	0.0202 (18)	0.040 (2)	−0.0052 (15)	0.0146 (18)	−0.0044 (16)
C7	0.0159 (17)	0.0197 (17)	0.037 (2)	−0.0009 (14)	0.0004 (15)	−0.0044 (15)
C8	0.0163 (17)	0.0185 (16)	0.0262 (17)	0.0007 (13)	−0.0024 (14)	−0.0016 (14)
C9	0.0206 (17)	0.0161 (15)	0.0188 (16)	0.0069 (13)	0.0071 (13)	0.0006 (13)
C10	0.0228 (18)	0.0118 (15)	0.0212 (16)	0.0025 (13)	0.0056 (13)	0.0009 (12)
C12	0.020 (2)	0.049 (3)	0.038 (2)	0.0017 (18)	0.0040 (17)	−0.0008 (19)
C13	0.036 (2)	0.053 (3)	0.039 (2)	0.017 (2)	0.0120 (19)	−0.005 (2)
C14	0.036 (2)	0.0224 (19)	0.037 (2)	−0.0001 (17)	−0.0006 (18)	−0.0047 (16)
C15	0.041 (2)	0.042 (2)	0.027 (2)	−0.0163 (19)	0.0094 (18)	0.0057 (17)
C16	0.054 (3)	0.034 (2)	0.032 (2)	−0.004 (2)	−0.004 (2)	−0.0119 (18)
C17	0.033 (2)	0.044 (2)	0.0229 (18)	0.0091 (19)	0.0058 (16)	0.0110 (17)
C18	0.032 (2)	0.049 (3)	0.0256 (19)	0.0172 (19)	0.0033 (17)	0.0146 (17)
C19	0.024 (2)	0.0237 (19)	0.050 (2)	0.0020 (16)	0.0091 (18)	0.0023 (17)

### Geometric parameters (Å, º)

**Table:**

Cu1—O2	1.979 (2)	C3—C4	1.384 (5)
Cu1—O3	1.981 (2)	C3—H3	0.9500
Cu1—N2	1.994 (3)	C4—C9	1.392 (4)
Cu1—N1	1.998 (3)	C4—H4	0.9500
Cu1—O4	2.365 (2)	C5—C6	1.371 (5)
Cu1—O1	2.394 (2)	C5—C10	1.383 (4)
S1—O1	1.517 (2)	C5—H5	0.9500
S1—C12	1.788 (4)	C6—C7	1.389 (5)
S1—C13	1.791 (4)	C6—H6	0.9500
S2—O2	1.536 (2)	C7—C8	1.384 (5)
S2—C14	1.771 (4)	C7—H7	0.9500
S2—C15	1.779 (4)	C8—H8	0.9500
S3—O3	1.531 (2)	C9—C10	1.488 (5)
S3—C16	1.772 (4)	C12—H12A	0.9800
S3—C17	1.775 (4)	C12—H12B	0.9800
S4—O4	1.512 (2)	C12—H12C	0.9800
S4—C19	1.785 (4)	C13—H13A	0.9800
S4—C18	1.786 (4)	C13—H13B	0.9800
Cl1—O6	1.419 (3)	C13—H13C	0.9800
Cl1—O5	1.430 (3)	C14—H14A	0.9800
Cl1—O8	1.431 (3)	C14—H14B	0.9800
Cl1—O7	1.435 (3)	C14—H14C	0.9800
Cl2_1—O9_1	1.388 (5)	C15—H15A	0.9800
Cl2_1—O11_1	1.413 (7)	C15—H15B	0.9800
Cl2_1—O12_1	1.421 (6)	C15—H15C	0.9800
Cl2_1—O10_1	1.436 (6)	C16—H16A	0.9800
Cl2_2—O9_2	1.399 (9)	C16—H16B	0.9800
Cl2_2—O12_2	1.413 (8)	C16—H16C	0.9800
Cl2_2—O10_2	1.421 (10)	C17—H17A	0.9800
Cl2_2—O11_2	1.432 (9)	C17—H17B	0.9800
N1—C1	1.340 (4)	C17—H17C	0.9800
N1—C9	1.351 (4)	C18—H18A	0.9800
N2—C8	1.343 (4)	C18—H18B	0.9800
N2—C10	1.352 (4)	C18—H18C	0.9800
C1—C2	1.398 (5)	C19—H19A	0.9800
C1—H1	0.9500	C19—H19B	0.9800
C2—C3	1.377 (5)	C19—H19C	0.9800
C2—H2	0.9500		
			
O2—Cu1—O3	94.62 (9)	C6—C5—C10	119.1 (3)
O2—Cu1—N2	174.98 (10)	C6—C5—H5	120.5
O3—Cu1—N2	90.37 (10)	C10—C5—H5	120.5
O2—Cu1—N1	93.53 (10)	C5—C6—C7	119.5 (3)
O3—Cu1—N1	170.76 (10)	C5—C6—H6	120.3
N2—Cu1—N1	81.45 (11)	C7—C6—H6	120.3
O2—Cu1—O4	87.42 (8)	C8—C7—C6	118.7 (3)
O3—Cu1—O4	90.21 (9)	C8—C7—H7	120.6
N2—Cu1—O4	92.05 (9)	C6—C7—H7	120.6
N1—Cu1—O4	85.81 (9)	N2—C8—C7	122.2 (3)
O2—Cu1—O1	86.15 (8)	N2—C8—H8	118.9
O3—Cu1—O1	94.52 (9)	C7—C8—H8	118.9
N2—Cu1—O1	93.98 (9)	N1—C9—C4	121.6 (3)
N1—Cu1—O1	90.35 (9)	N1—C9—C10	114.9 (3)
O4—Cu1—O1	172.30 (8)	C4—C9—C10	123.5 (3)
O1—S1—C12	106.68 (17)	N2—C10—C5	122.1 (3)
O1—S1—C13	104.87 (17)	N2—C10—C9	113.9 (3)
C12—S1—C13	98.2 (2)	C5—C10—C9	124.0 (3)
O2—S2—C14	104.83 (16)	S1—C12—H12A	109.5
O2—S2—C15	102.73 (17)	S1—C12—H12B	109.5
C14—S2—C15	99.78 (19)	H12A—C12—H12B	109.5
O3—S3—C16	105.11 (17)	S1—C12—H12C	109.5
O3—S3—C17	103.87 (15)	H12A—C12—H12C	109.5
C16—S3—C17	99.7 (2)	H12B—C12—H12C	109.5
O4—S4—C19	107.37 (15)	S1—C13—H13A	109.5
O4—S4—C18	106.68 (16)	S1—C13—H13B	109.5
C19—S4—C18	97.6 (2)	H13A—C13—H13B	109.5
S1—O1—Cu1	120.43 (13)	S1—C13—H13C	109.5
S2—O2—Cu1	119.24 (13)	H13A—C13—H13C	109.5
S3—O3—Cu1	125.31 (14)	H13B—C13—H13C	109.5
S4—O4—Cu1	142.59 (14)	S2—C14—H14A	109.5
O6—Cl1—O5	110.0 (2)	S2—C14—H14B	109.5
O6—Cl1—O8	109.9 (2)	H14A—C14—H14B	109.5
O5—Cl1—O8	109.46 (18)	S2—C14—H14C	109.5
O6—Cl1—O7	109.21 (19)	H14A—C14—H14C	109.5
O5—Cl1—O7	109.5 (2)	H14B—C14—H14C	109.5
O8—Cl1—O7	108.8 (2)	S2—C15—H15A	109.5
O9_1—Cl2_1—O11_1	109.7 (4)	S2—C15—H15B	109.5
O9_1—Cl2_1—O12_1	112.2 (3)	H15A—C15—H15B	109.5
O11_1—Cl2_1—O12_1	106.6 (4)	S2—C15—H15C	109.5
O9_1—Cl2_1—O10_1	109.6 (3)	H15A—C15—H15C	109.5
O11_1—Cl2_1—O10_1	107.4 (4)	H15B—C15—H15C	109.5
O12_1—Cl2_1—O10_1	111.2 (4)	S3—C16—H16A	109.5
O9_2—Cl2_2—O12_2	112.9 (7)	S3—C16—H16B	109.5
O9_2—Cl2_2—O10_2	109.9 (7)	H16A—C16—H16B	109.5
O12_2—Cl2_2—O10_2	115.2 (8)	S3—C16—H16C	109.5
O9_2—Cl2_2—O11_2	108.1 (7)	H16A—C16—H16C	109.5
O12_2—Cl2_2—O11_2	104.7 (7)	H16B—C16—H16C	109.5
O10_2—Cl2_2—O11_2	105.4 (7)	S3—C17—H17A	109.5
C1—N1—C9	119.4 (3)	S3—C17—H17B	109.5
C1—N1—Cu1	126.2 (2)	H17A—C17—H17B	109.5
C9—N1—Cu1	114.4 (2)	S3—C17—H17C	109.5
C8—N2—C10	118.5 (3)	H17A—C17—H17C	109.5
C8—N2—Cu1	126.3 (2)	H17B—C17—H17C	109.5
C10—N2—Cu1	115.0 (2)	S4—C18—H18A	109.5
N1—C1—C2	121.5 (3)	S4—C18—H18B	109.5
N1—C1—H1	119.2	H18A—C18—H18B	109.5
C2—C1—H1	119.2	S4—C18—H18C	109.5
C3—C2—C1	119.2 (3)	H18A—C18—H18C	109.5
C3—C2—H2	120.4	H18B—C18—H18C	109.5
C1—C2—H2	120.4	S4—C19—H19A	109.5
C2—C3—C4	119.4 (3)	S4—C19—H19B	109.5
C2—C3—H3	120.3	H19A—C19—H19B	109.5
C4—C3—H3	120.3	S4—C19—H19C	109.5
C3—C4—C9	118.9 (3)	H19A—C19—H19C	109.5
C3—C4—H4	120.5	H19B—C19—H19C	109.5
C9—C4—H4	120.5		
			
C12—S1—O1—Cu1	−85.9 (2)	O1—Cu1—N1—C9	98.2 (2)
C13—S1—O1—Cu1	170.58 (18)	O2—Cu1—N2—C8	−179 (55)
O2—Cu1—O1—S1	24.70 (16)	O3—Cu1—N2—C8	−4.9 (3)
O3—Cu1—O1—S1	−69.64 (17)	N1—Cu1—N2—C8	179.4 (3)
N2—Cu1—O1—S1	−160.33 (17)	O4—Cu1—N2—C8	−95.1 (3)
N1—Cu1—O1—S1	118.21 (17)	O1—Cu1—N2—C8	89.7 (3)
O4—Cu1—O1—S1	58.2 (7)	O2—Cu1—N2—C10	−3.6 (12)
C14—S2—O2—Cu1	−109.80 (17)	O3—Cu1—N2—C10	170.5 (2)
C15—S2—O2—Cu1	146.32 (17)	N1—Cu1—N2—C10	−5.2 (2)
O3—Cu1—O2—S2	−42.44 (15)	O4—Cu1—N2—C10	80.2 (2)
N2—Cu1—O2—S2	131.6 (11)	O1—Cu1—N2—C10	−95.0 (2)
N1—Cu1—O2—S2	133.21 (15)	C9—N1—C1—C2	0.4 (5)
O4—Cu1—O2—S2	47.57 (14)	Cu1—N1—C1—C2	−177.8 (2)
O1—Cu1—O2—S2	−136.67 (15)	N1—C1—C2—C3	0.8 (5)
C16—S3—O3—Cu1	−106.8 (2)	C1—C2—C3—C4	−1.3 (5)
C17—S3—O3—Cu1	148.92 (19)	C2—C3—C4—C9	0.7 (5)
O2—Cu1—O3—S3	−41.26 (17)	C10—C5—C6—C7	−0.3 (5)
N2—Cu1—O3—S3	139.26 (17)	C5—C6—C7—C8	0.5 (5)
N1—Cu1—O3—S3	166.9 (5)	C10—N2—C8—C7	−0.9 (5)
O4—Cu1—O3—S3	−128.69 (16)	Cu1—N2—C8—C7	174.3 (2)
O1—Cu1—O3—S3	45.24 (17)	C6—C7—C8—N2	0.1 (5)
C19—S4—O4—Cu1	−49.5 (3)	C1—N1—C9—C4	−1.1 (4)
C18—S4—O4—Cu1	54.3 (3)	Cu1—N1—C9—C4	177.3 (2)
O2—Cu1—O4—S4	−133.1 (2)	C1—N1—C9—C10	179.0 (3)
O3—Cu1—O4—S4	−38.5 (2)	Cu1—N1—C9—C10	−2.7 (3)
N2—Cu1—O4—S4	51.9 (2)	C3—C4—C9—N1	0.5 (5)
N1—Cu1—O4—S4	133.1 (2)	C3—C4—C9—C10	−179.5 (3)
O1—Cu1—O4—S4	−166.6 (5)	C8—N2—C10—C5	1.1 (5)
O2—Cu1—N1—C1	2.6 (3)	Cu1—N2—C10—C5	−174.6 (2)
O3—Cu1—N1—C1	154.5 (5)	C8—N2—C10—C9	−179.1 (3)
N2—Cu1—N1—C1	−177.5 (3)	Cu1—N2—C10—C9	5.2 (3)
O4—Cu1—N1—C1	89.8 (3)	C6—C5—C10—N2	−0.5 (5)
O1—Cu1—N1—C1	−83.5 (3)	C6—C5—C10—C9	179.7 (3)
O2—Cu1—N1—C9	−175.6 (2)	N1—C9—C10—N2	−1.6 (4)
O3—Cu1—N1—C9	−23.7 (7)	C4—C9—C10—N2	178.4 (3)
N2—Cu1—N1—C9	4.2 (2)	N1—C9—C10—C5	178.1 (3)
O4—Cu1—N1—C9	−88.5 (2)	C4—C9—C10—C5	−1.8 (5)

### Hydrogen-bond geometry (Å, º)

**Table:**

*D*—H···*A*	*D*—H	H···*A*	*D*···*A*	*D*—H···*A*
C1—H1···O2	0.95	2.52	3.046 (4)	115
C6—H6···O8^i^	0.95	2.47	3.255 (5)	140
C8—H8···O3	0.95	2.42	2.946 (4)	115
C4—H4···O1^ii^	0.95	2.35	3.219 (4)	151
C16—H16*B*···O5^iii^	0.98	2.56	3.411 (5)	145
C17—H17*B*···O5^iii^	0.98	2.40	3.306 (5)	153
C15—H15*C*···O7^iv^	0.98	2.51	3.320 (5)	140

Symmetry codes: (i) −*x*+1, −*y*+1, −*z*; (ii) −*x*, −*y*+1, −*z*; (iii) *x*−1/2, −*y*+1/2, *z*+1/2; (iv) −*x*, −*y*, −*z*.
